# A Multicenter Trial Defining a Serum Protein Signature Associated with Pancreatic Ductal Adenocarcinoma

**DOI:** 10.1155/2015/587250

**Published:** 2015-10-26

**Authors:** Anna S. Gerdtsson, Núria Malats, Anna Säll, Francisco X. Real, Miquel Porta, Petter Skoog, Helena Persson, Christer Wingren, Carl A. K. Borrebaeck

**Affiliations:** ^1^Department of Immunotechnology and CREATE Health, Lund University, Medicon Village 406, 223 81 Lund, Sweden; ^2^Genetic and Molecular Epidemiology Group, Spanish National Cancer Research Centre (CNIO), C/Melchor Fernández Almagro 3, 28029 Madrid, Spain; ^3^Epithelial Carcinogenesis Group, Spanish National Cancer Research Centre (CNIO), C/Melchor Fernández Almagro 3, 28029 Madrid, Spain; ^4^Department of Experimental and Health Sciences, University of Pompeu Fabra, Dr. Aiguader 88, 08003 Barcelona, Spain; ^5^Hospital del Mar Medical Research Institute (IMIM), CIBERESP, UAB, Dr. Aiguader 88, 08003 Barcelona, Spain

## Abstract

*Background. *Pancreatic ductal adenocarcinoma (PDAC) is an aggressive disease with rapid tumor progression and poor prognosis. This study was motivated by the lack of sensitive and specific PDAC biomarkers and aimed to identify a diagnostic, serum protein signature for PDAC. *Methods. *To mimic a real life test situation, a multicenter trial comprising a serum sample cohort, including 338 patients with either PDAC or other pancreatic diseases (OPD) and controls with nonpancreatic conditions (NPC), was analyzed on 293-plex recombinant antibody microarrays targeting immunoregulatory and cancer-associated antigens. *Results.* Serum samples collected from different hospitals were analyzed and showed that (i) sampling from five different hospitals could not be identified as a preanalytical variable and (ii) a multiplexed biomarker signature could be identified, utilizing up to 10 serum markers that could discriminate PDAC from controls, with sensitivities and specificities in the 91–100% range. The first protein profiles associated with the location of the primary tumor in the pancreas could also be identified. *Conclusions. *The results demonstrate that robust enough serum signatures could be identified in a multicenter trial, potentially contributing to the development of a multiplexed biomarker immunoassay for improved PDAC diagnosis.

## 1. Introduction

Pancreatic ductal adenocarcinoma (PDAC) is the 4th most common cancer-related cause of death [1]. Multiple factors account for its poor prognosis and improved diagnosis provides today the only possibility for cure. PDAC is often detected at late stages with 80% of patients not eligible for surgery due to either locally advanced or metastatic disease [1–3]. Genetic data suggest a time frame of at least 5 years from tumor initiation until the acquisition of metastatic ability [4], thus pointing towards a window of opportunity for detection if markers were available.

The most extensively evaluated marker for PDAC, CA19-9, suffers from poor specificity and the use of CA19-9 alone for PDAC screening has been discouraged [5]. In recent years, the field has moved towards multiplexed marker panels in search of increased sensitivity and specificity [6]. The protein panels assessed have commonly consisted of 2–5 analytes mainly selected on the basis of literature searches, or following mass spectrometry-based discovery studies, and measured in serial or in parallel using ELISA or equivalent methods [7, 8]. The markers have primarily been highly abundant blood proteins, acute-phase reactants (e.g., CRP and SAA), tumor markers (e.g., CA242, CA125, and CEA), adhesion molecules (e.g., ICAM-1 and ADCAM), proteins involved in extracellular matrix degradation (e.g., MMPs and TIMP1), and lipoproteins (e.g., Apo-C1, Apo-A2), most often in combination with CA19-9 [9–14].

Given the inflammatory mechanisms of cancer, the immunoregulatory serum proteome is also a relevant source of potential cancer biomarkers [15]. However, systemic effects and the multitude of functions of many proteins of the immune system suggest that panels of 2–5 markers might not be sufficiently specific for PDAC, particularly for the differential diagnosis with chronic pancreatitis (CP) and other conditions sharing symptoms and/or mechanisms. In fact, previous studies have shown that an increased number of immunoregulatory proteins (≤25) may yield highly disease-specific signatures [16–18]. Nevertheless, analysis of the immunoregulatory proteome faces several challenges. First, the serum concentration of proteins of interest displays a vast dynamic range, from high microgram to low picogram per mL, complicating their simultaneous detection, using conventional proteomic methodologies [19, 20]. Second, promising cancer markers are more likely to be found among the low abundant, often low-molecular weight, proteins, which so far can only be targeted using affinity proteomics with high-performing binders [21, 22]. Third, disease-associated changes in serum levels of low-abundance analytes are expected to be small, requiring a large number of samples for a statistically significant demonstration of benefit [23].

For these purposes, we have designed microarrays with close to 300 human recombinant scFv antibodies. Originally, this platform mainly targeted the immunoregulatory proteome [24] but has now been extended with a set of novel antibodies selected against predominantly cancer-associated antigens. With these highly multiplexed arrays, protein expression can be measured in hundreds of samples in a reproducible and high-throughput manner. Moreover, this discovery mode platform could be straightforwardly scaled down to a disease-specific biomarker assay for clinical application. The aim of the current study was to perform a multicenter trial, where serum collected at several different sites was analyzed, and to identify a robust, diagnostic serum protein signature associated with PDAC at time of clinical diagnosis.

## 2. Materials and Methods

### 2.1. Samples

This retrospective study analyzed 338 serum samples from patients with PDAC (*n* = 156) and other pancreatic diseases (OPD) (*n* = 152) and normal pancreatic controls (NPC) (*n* = 30) that were collected after local ethical approval and informed consent at five different hospitals in Spain (Hospital del Mar, Barcelona; Hospital Vall Hebron, Barcelona; Hospital Mútua de Terrassa, Terrassa; Hospital Son Dureta, Palma de Mallorca; Hospital General Universitario de Elche, Elche), as part of the PANKRAS-II Study [25, 26] from 1992 to 1995 (Table 1).

The study included patients with a suspicion of PDAC managed in the participating hospitals, and one sample was drawn from each patient, using standardized protocols. A panel of experts validated by consensus the final diagnosis of all patients through a careful revision of clinical and pathological records and follow-up information [27]. We have previously shown that PDAC can be accurately discriminated from healthy volunteers, using a smaller serum cohort and an earlier version of the antibody microarray platform [17], which is why we in the current study focused on pancreatitis as the main control group. The smaller group of NPC patients included for reference was mainly attended to in the services of general surgery and digestive and traumatology of the participant hospitals, mostly including orthopedic fractures and hernias (Table 1, footnote). Samples were collected before any treatment was given, separated within 3 h and stored as 1 mL aliquots at −80°C. The entire set of samples was labeled at a single occasion, using a previously optimized protocol [28, 29]. Briefly, crude samples were diluted 1 : 45 in PBS, resulting in an approximate protein concentration of 2 mg/mL, and labeled with a 15 : 1 molar excess of biotin to protein, using 0.6 mM EZ-Link Sulfo-NHS-LC-Biotin (Pierce, Rockford, IL, USA). Unbound biotin was removed by dialysis using a 3.5 kDa MW dialysis membrane (Spectrum Laboratories, Rancho Dominguez, CA, USA) against PBS for 72 hours, with a change of buffer every 24 hours. Labeled samples were aliquoted and stored at −20°C.

### 2.2. Antibodies

The antibody microarrays contained 293 human recombinant scFv antibodies directed against 98 known antigens (Supplementary Table 1 in Supplementary Material available online at http://dx.doi.org/10.1155/2015/587250) and 31 peptides motifs (Supplementary Table 2) [30]. Most antibodies were selected against immunoregulatory proteins and have previously demonstrated robust on-chip functionality [31–33]. Several binders have also been validated, using ELISA, mass spectrometry, and spiking and/or blocking experiments (Supplementary Table 1). In addition, 76 scFvs targeting 28 additional antigens were selected from the Hell-11 phage display library (Säll et al., manuscript in preparation) against predominantly cancer-associated targets, including kinases and other enzymes, transcriptional regulators, cytokines, and receptors. Although these binders have not previously been used in microarray applications, their on-chip functionality has been demonstrated in an independent study (Säll et al., manuscript in preparation). The antibodies were produced in* E. coli* and purified from the periplasm, using a MagneHis Protein Purification System (Promega, Madison, WI, USA). The elution buffer was exchanged for PBS, using Zeba 96-well desalt spin plates (Pierce). The protein yield was measured using NanoDrop (Thermo Scientific, Wilmington, DE, USA) and the purity was checked using 10% SDS-PAGE (Invitrogen, Carlsbad, CA, USA).

### 2.3. Antibody Microarrays

Antibody microarrays were produced on black MaxiSorp slides (NUNC, Roskilde, Denmark), using a noncontact printer (SciFlexarrayer S11, Scienion, Berlin, Germany). Thirteen identical subarrays were printed on each slide, each array consisting of 33 × 31 spots (130 *μ*m spot diameter) with 200 *μ*m spot-to-spot center distance. Each subarray consisted of 3 segments, separated by rows of labeled BSA (Supplementary Figure 1) and each antibody was printed in 3 replicates, one in each segment and in different segment positions for each replicate. For each round of analysis, 8 slides (104 arrays) were printed overnight and the slides were used for array analysis the following day. All samples were blindly analyzed over the course of 5 consecutive days.

Each slide was mounted in a hybridization gasket (Schott, Jena, Germany) and blocked with PBSMT (1% (w/v) milk, 1% (v/v) Tween-20 in PBS) for 1 h. Meantime, aliquots of labeled serum samples were thawed on ice and diluted 1 : 10 in PBSMT. The slides were washed 4 times with PBST (0.05% (v/v) Tween-20 in PBS) before 120 *μ*L of the samples were added. Samples were incubated for 2 h on a rocking table and slides were washed 4 times with PBST, incubated with 1 *μ*g/mL Streptavidin-Alexa in PBSMT for 1 h on a rocking table, and again washed 4 times with PBST. Finally, the slides were dismounted from the hybridization chambers, quickly immersed in dH_2_O, and dried under a stream of N_2_. The slides were immediately analyzed, using a confocal microarray scanner (PerkinElmer Life and Analytical Sciences, Wellesley, MA, USA) at 10 *μ*m resolution, using 60% PMT gain and 90% laser power. Signal intensities were quantified, using the ScanArray Express software version 4.0 (PerkinElmer Life and Analytical Sciences) with the fixed circle option. After local background subtraction, intensity values were used for data analysis. Data acquisition was performed by a trained member of the research team blinded to the sample classification and clinical data.

### 2.4. Data Preprocessing

An average of the 3 replicate spots was used, unless any replicate CV exceeded 15% from the mean value, in which case it was dismissed and the average of the 2 remaining replicates was used instead. The average CV of replicates was 8.3% (±5.5%). Applying a cut-off CV of 15%, 70% of data values were calculated from all 3 replicates and the remaining 30% from 2 replicates.

For evaluation of normalization strategies and data distribution, the data was visualized using 3D principal component analysis (PCA) with ANOVA filtering (Qlucore AB, Lund, Sweden). Two samples (OPD) were excluded as barely any signals were obtained from them for reasons that were not further explored. Of note, ANOVA on log10 raw data showed no significant (*p* < 0.01) differences between (i) sample subarray positioning on slide, (ii) patient gender, (iii) patient age, and (iv) participating clinical center (data not shown). Minor systematic differences were observed between days of analysis (rounds 1–5, likely due to small differences in humidity during array printing, in particular for day 1; see Supplementary Figure 2(A)), which could be neutralized by normalization (Supplementary Figure 2(B)). The data was normalized in two steps. First, differences between rounds (days) of analysis were eliminated, using a subtract group mean strategy [34]. The average intensity from each antibody was calculated within each round of analysis and subtracted from the single values, thus zero-centering the data. The global mean signal from each antibody was added to each respective data point to avoid negative values. Second, array-to-array differences (e.g., inherent sample background fluorescence differences (see Supplementary Figure 1)) were handled by calculating a scaling factor for each subarray, based on the 20% of antibodies with the lowest CV, as has been previously described [17, 35].

### 2.5. Data Analysis

Two-group comparisons (PDAC versus NPC and PDAC versus OPD) were performed using PCA, Student's *t*-test, Benjamini Hochberg procedure for false discovery rate control (*q*-values), and fold changes. A group ANOVA was also performed (Qlucore). SVM analysis was performed in R, using a linear kernel with the cost of constraints set to 1. For SVM analyses, the data was randomly divided into training and test sets with 2/3 of the samples from each group as training set and the remaining samples as test set. A backward elimination algorithm was applied, using training set data, excluding one antibody at the time, and iteratively eliminating the antibody that was removed when the smallest Kullback-Leibler divergence was obtained in the classification analysis, as previously described [36]. The last 25 antibodies to be eliminated were used to build a classification model in the training set, which then was frozen and applied in the corresponding independent test set, a procedure that reduces overfitting of data. The area under the ROC-curve (AUC) was used as a measure of the accuracy of performance of the signature in the test set. This procedure was repeated 10 times, in 10 different and randomly generated pairs of training and test sets. Ultimately, each antibody was given a score based on the order of elimination in the 10 training sets. The score was calculated from the average endurance in the elimination process (first antibody to be eliminated: 1; last antibody to be eliminated: 293). Sensitivities (SN) and specificities (SP) and positive (PPV) and negative (NPV) predictive values were calculated from SVM prediction value threshold of zero. Finally, pathway analyses were performed, using MetaCore (Thomson Reuters, New York, NY, USA).

## 3. Results

### 3.1. Differential Protein Expression Analysis

**Figure 1:**
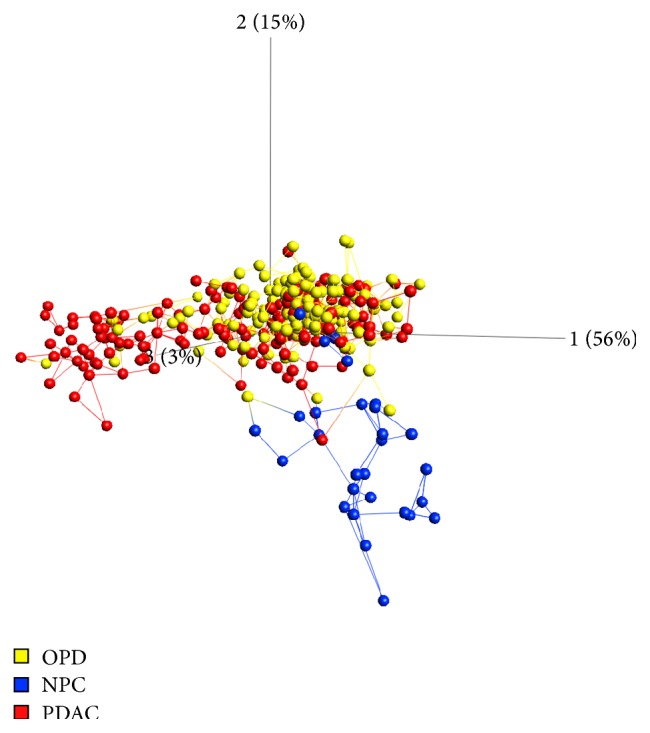
Principal component analysis with samples colored according to diagnosis (red: PDAC; yellow: OPD; blue: NPC). The data was filtered to *p* < 1*E* − 10 (63 antibodies).

Differential expression analysis was performed as a measure of biological differences of individual proteins in samples representing cancer (PDAC) and controls (NPC), respectively. The analysis revealed a number of proteins with strong differential expression patterns and a multigroup ANOVA showed that 75% of the markers displayed a level of significance with *p* < 0.001. A PCA indicated that the PDAC samples differed more from NPC than from OPD (Figure 1), the latter containing mostly various inflammatory states of pancreas. For each subgroup comparison, the 25 protein markers displaying the highest level of discrimination are shown in Table 2. The markers with the highest differential expression were GAK, IL-6, LDL, and MAPK8 for PDAC versus NPC and Cystatin C, IL-13, and IL-1*α* for PDAC versus OPD.

### 3.2. Protein Signature for PDAC Classification

**Figure 2:**
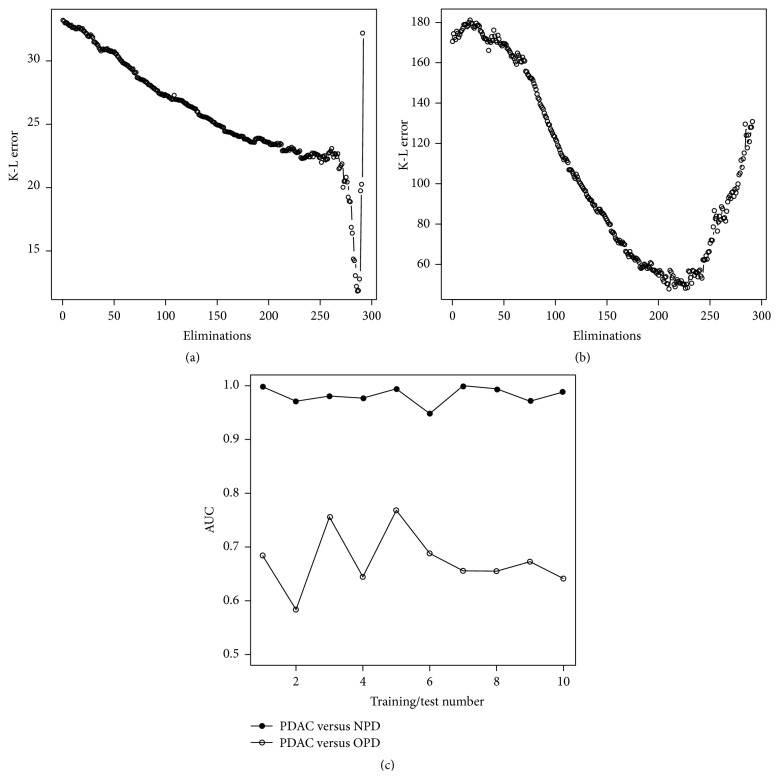
Backward elimination filtration. The Kullback-Leibler (K-L) error after each round of antibody elimination in the first training sets was plotted for (a) PDAC versus NPC; (b) PDAC versus OPD; and (c) AUC values generated from the 25-antibody SVM models from 10 different pairs of training/test sets.

Differential protein expression analysis can produce valuable information from a biological point of view. Importantly, discriminatory biomarker signatures cannot however be optimally derived from individual *p* values or fold changes, since these values do not necessarily represent orthogonal information. To circumvent this, we have developed a backward elimination algorithm, which eliminates markers providing similar information and that identifies marker combinations representing the highest predictive power. To avoid overinterpretation and to demonstrate robustness of the data set, it was randomly divided into training and test sets, and the SVM-based backward elimination algorithm was then applied in the training sets. The classification of PDAC versus controls (NPC) was highly accurate, as implied by small Kullback-Leibler (K-L) divergences (≤33.2) throughout the elimination process (Figure 2(a)). In the first training set, a distinct K-L minimum (12.0) was reached when only 7 markers remained in the elimination process. This 7-plex protein panel, including IL-6, Cystatin C, IL-8, IL-11, C1 inhibitor, Eotaxin, and HADH2, displayed a SN and PPV of 98% and SP and NPV of 90%, when subsequently applied in a test set of independent samples. This demonstrates that a handful of markers can be combined into a signature, displaying a highly accurate classification of PDAC versus NPC controls. In contrast, the K-L values were higher (≤181.3) when PDAC was compared to OPD. Here, the minimum K-L value (50.0) was not as distinct, which could be compensated with a larger panel of markers for optimal differentiation (Figure 2(b)). For each comparison, the procedure above was repeated until 10 different, randomly generated training sets had been used for backward elimination (data not shown). The resulting set of K-L curves were highly similar to those shown in Figure 2, indicating that a signature of 4–10 antibodies would be sufficient for PDAC versus NPC classification (Figure 2(a)), while an average of 67 antibodies were necessary for optimal classification of PDAC versus OPD (Figure 2(b)).

Based on previous data [16–18], the top 25 antibodies from each elimination process were selected for the purpose of evaluating a mid-size signature and used to build SVM classification models in the training sets. The AUC values generated in the independent test sets were used as a measure of the classification accuracy (Figure 2(c)). Each signature could discriminate PDAC from NPC controls with high accuracy (average AUC 0.98). The sensitivity and specificity of the ten signatures displayed an average of 99% SN, 80% SP. The corresponding predictive values ranged from 93% PPV, 86% NPV to 100% PPV, 100% NPV, with an average PPV of 96% and NPV of 95%. Despite the fact of the more heterogeneous composition of the OPD cohort, still a discrimination, although less accurate, between PDAC and OPD could be identified (average AUC 0.7), with 62% SN, 80% SP and 73% PPV, 71% NPV. Finally, each antibody was given a score, corresponding to its average performance in the elimination processes (Table 3).

The 10 signatures from the PDAC versus NPC analysis were highly similar. For example, the top antibody, targeting IL-11, had an overall score of 291.4 out of 293 eliminations, that is, being the last antibody to be eliminated 4 out of 10 times. In all, the 25 highest scored antibodies for PDAC versus NPC represented 20 nonredundant markers, including cytokines and chemokines (IL-11, IL-6, IL-13, IL-8, TNF-*α*, and Eotaxin), complement components (C1 inhibitor, C1q, C5, and Factor B), and enzymes (HADH2, GAK, and ATP-5B). As expected, a much different set of proteins appeared as top markers for PDAC versus OPD, with MAPK1, TNFRSF3, UCHL5, IL-4, Apo-A1, Apo-A4, CD40 ligand, and KSYK, among the top scored analytes (Table 3). As could be expected, the signatures derived by backward elimination (antibody score, Table 3) were different from those derived from differential expression analysis (Table 2), although some overlap was observed, particularly for the PDAC versus NPC signature.

### 3.3. Tumor Site Location

**Figure 3:**
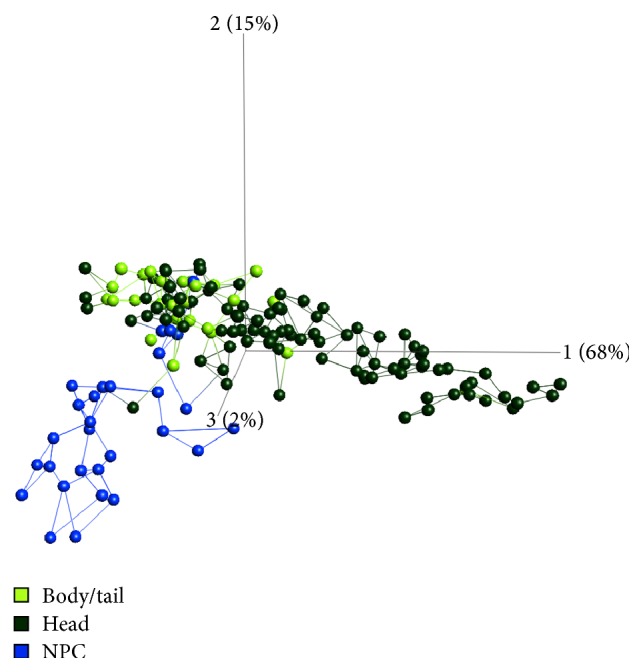
Principal component analysis with samples colored according to tumor localization (light green: body and tail tumors; dark green: head tumors; blue: NPC). The data was filtered to *p* < 1*E* − 10 (46 antibodies).

The serum samples could also be discriminated depending on the location of the primary tumor in the pancreas. PCA indicated that patients with tumors located in the body or the tail of the pancreas clustered closer to NPC subjects compared to patients with tumors in the head of the pancreas (Figure 3). Of note, protein markers in samples derived from patients with a tumor location in the head of pancreas could still discriminate body/tail tumor samples versus NPC, indicating that the general PDAC signature is not affected by tumor site. The differential protein expression analysis also revealed an extensive list of (different) markers in the intrapancreatic comparison of head versus body/tail tumors, with 39% of the markers displaying *p* values < 0.001, almost exclusively upregulated levels in serum from head tumors compared to body/tail tumor samples (Supplementary Table 3).

## 4. Discussion

To demonstrate the robustness of the multiplexed approach for cancer detection, we have profiled 338 serum samples, using a large multicenter clinical cohort with subjects with a broad range of diagnoses [26]. The differential levels of many proteins are likely to correlate when using such highly multiplexed assay, particularly when measuring several interconnected proteins like those of the immune system. Even though the discriminative power of individual proteins, represented by single *p* values and *q*-values, might be of interest, it is important to realize that other approaches should be applied to identify the optimal combination of markers [37]. Here we used a supervised model based on Support Vector Machine analysis in combination with a backward elimination algorithm. The data was subdivided into training sets, from which biomarker signatures were identified. The classification power of these signatures was subsequently evaluated in separate test sets of the remaining samples. Using this approach, discriminative combinations of markers, that is, signatures for which each marker likely contributed with orthogonal information, were obtained. This analysis suggested that up to 10 protein markers were sufficient for a highly accurate discrimination of PDAC versus NPC controls. The results support our previous observations that PDAC patients can be accurately discriminated from healthy individuals by analyzing the serum proteome [17, 18]. It should be noted, however, that the normal pancreatic control group in this specific study were patients from the participating hospitals attending for other, nonpancreatic conditions, and it cannot be ruled out that some of these patients may have had an ongoing inflammatory response detectable in their serum proteome. For optimal classification accuracy of PDAC versus the larger control group of other pancreatic diseases, a much larger protein panel (*n* = 67) was required. The high number of markers needed could be due to shared pathophysiology of PDAC and CP (CP is a common comorbidity of PDAC) and shared histological changes (inflammation and fibrosis). Furthermore, greater clinical heterogeneity of the OPD group, compared to control groups used in previously analyzed cohorts [17, 18, 38], most probably explains why we did not reach 75/100% SN/SP, as reported earlier for CP.

A pathway analysis based on the entire set of antibodies with corresponding *p* values and fold changes for PDAC versus NPC demonstrated similar systemic impacts of PDAC, as compared to conditions such as hyperinsulinism and insulin resistance, as well as biomarkers associated with diabetes type I/II (Supplementary Figure 3). Even though the most significant pathways included the alternative complement pathway and different cytokine signaling pathways, the analysis still revealed disease and core biomarker networks, such as, for example, diabetes types I and II, and autoimmune and infectious conditions, some related to PDAC. This data emphasizes the challenge in distinguishing PDAC from symptomatically related benign conditions [18, 38] and also underlines the need for multiplexing to reach sufficient discrimination power.

Ongoing follow-up studies will show whether the signature identified here could be condensed to even smaller panels of markers, while maintaining the sensitivity and specificity required for a diagnostic immunoassay. As discussed above, we selected the top 25 antibodies from each elimination process as a starting point to build SVM classification models in training sets. The AUC values subsequently generated were used as a measure of classification accuracy, where AUC up to 0.98 was reached (PDAC versus NPC). Despite adopting two highly different strategies for signature identification (differential protein expression versus SVM-based backward elimination), there was still a substantial overlap between the signatures; that is, the markers with the highest overall backward elimination scores were also significantly differentially expressed. For example, IL-11, IL-6, C1 inhibitor, IL-13, HADH2, LDL, GAK, C1q, and TNF-*α* appeared in both signatures and, thus, demonstrated both low *p* values and *q*-values as well as high backward elimination scores for PDAC versus NPC. For PDAC versus OPD the two signatures overlapped by C5, Apo-A4, BTK, TGF-*β*1, MCP-1, and UPF3B. Of note, a large number of markers that have been associated with PDAC in previous studies were identified in the present comparisons with both NPC and OPD, including C1 inhibitor, C5, Factor B, IL-13, MCP-1, and TNF-*α* [17, 18, 39–41]. In addition, new potential PDAC markers were identified, illustrated by HADH2, a 3-hydroxyacyl-CoA dehydrogenase type II, and the serine/threonine kinase GAK (Cyclin G associated kinase), a direct transcriptional target of p53. GAK was markedly downregulated in PDAC versus NPC and also appeared in the backward elimination signature for PDAC versus NPC. In addition, TNFRSF3 (a TNF-*β* receptor) and UPF3B (an mRNA regulatory protein) were included in both the differential expression analysis and backward elimination score s for PDAC versus OPD. Finally, MAPK1 (ERK2), a kinase of the MAPK/ERK signaling pathway which is deregulated in PDAC and other cancers [42], was the highest scoring protein in the backward elimination signatures for PDAC versus OPD. Of note, these proteins display diverse subcellular distributions and some, such as GAK, HADH2, MAPK1, and TNFRSF3, to the best of our knowledge have not previously been identified in serum.

A new finding in this study was the observation that serum protein markers associated with tumor localization were identified. A major problem with tumors of the body/tail in comparison with pancreatic head cancer is distant metastasis, especially in the liver, and resection of the tumor does not increase postoperative survival in metastatic disease [43]. On the other hand, patients with local-stage body/tail tumors had higher survival rates compared with local-stage pancreatic head cancer [44]. Our data indicated markers in samples from patients with body/tail tumors clustering closer to the NPC controls, as compared to samples from patients with pancreatic head tumors. This may be explained by a more profound systemic impact of the head tumors, as these are prone to invade the surrounding mesenteric blood vessels connecting the pancreas to the duodenum [2], or by changes secondary to biliary obstruction. As the biological differences can result in different treatment efficiency [43], biomarkers that can discriminate between tumor localization could be of clinical relevance.

Despite being the 4th most lethal cancer, the incidence of PDAC is low with ~11 per 100 000 individuals in the US [45]. Given the increasing mortality in Europe and USA, PDAC will likely become a more serious problem over the next decades [46, 47]. Still, it might be argued that the low incidence does not justify screening for PDAC in the general population. In light of this, a recent health-economic study based on sensitivities and specificities presented by us from a previous trial [18] suggests the cost-effectiveness of screening for PDAC in high-risk groups, such as patients with familiar history of PDAC chronic pancreatitis, Peutz-Jeghers syndrome, and newly onset diabetes mellitus [48, 49]. PDAC patients in the current cohort had predominantly stage III and IV tumors, and consequently studies have been initiated to explore the diagnostic power of the marker signature in patients with stage I and II tumors, as well as subjects from the relevant risk groups.

## 5. Conclusion

In summary, this multicenter study allowed identification of protein signatures associated with PDAC, displaying sensitivities and specificities in the 91–100% range, demonstrating that different sites for sample collection were not a confounding factor affecting the robustness of the signature. Furthermore, we provide an indication that recombinant antibody microarrays could identify serum protein markers associated with different tumor locations in the pancreas, although this observation and its clinical implications need to be further corroborated. Further refinement of the protein patterns defined in this study and previous cohorts and validation in additional sample sets could lead to biomarker signatures providing improved ability for diagnosis of this disease with such dismal prognosis.

## Supplementary Material

Supplementary Figure 1: Slide and microarray lay-out.
Supplementary Figure 2: Data visualized by principal component analysis.
Supplementary Figure 3: Biomarker assessment by database queries for diseases by biomarkers.
Supplementary Table 1: Antigens targeted on the antibody microarray.
Supplementary Table 2: Context-independent motif specific antibodies used on the antibody microarrays.

## Figures and Tables

**Table 1:** Characteristics of the PANKRAS II study subjects according to the groups considered in the comparative analysis.

Diagnosis	Number of samples	Gender (M/F)	Age (mean ± sd.)
Pancreatic cancer (PDAC)	**156**	**92/64**	**66 ± 13**
Head (ICD9: 157.0)	97	45/52	67 ± 13
Body (ICD9: 157.1)	16	12/4	68 ± 10
Tail (ICD9: 157.2)	10	8/2	60 ± 11
Other (ICD9: 157.8)	16	8/8	65 ± 13
Unspecified (ICD9: 157.9)	17	12/5	66 ± 15
Other pancreatic diseases (OPD)	**152**	**117/35**	**52 ± 14**
Acute pancreatitis (ICD9: 577.0)	33	22/11	59 ± 15
Chronic pancreatitis (ICD9: 577.1)	110	95/15	50 ± 13
Islet neoplasm (ICD9: 211.7)	3	0/3	64 ± 3
Benign pancreatic neoplasms (ICD9: 211.6)	6	0/6	51 ± 18
Nonpancreatic conditions (NPC)^*∗*^	**30**	**20/10**	**62 ± 14**

Total	**338**		

(*∗*) ICD9 codes: 454 (2), 540, 735 (2), 807, 808, 812, 813, 820, 550.9 (4), 553.21 (2), 553.3, 560.9, 603.9, 735.0, 806.4, 812.09, 815.02, 820.2, 820.8, 821.2, 823.0, 823.90, 824.0, 839.0.

**Table 2:** The top 25 differentially expressed analytes as derived from Student's *t*-test. The Benjamini Hochberg *q*-value is shown for each antibody. For antigens targeted by multiple clones, the individual antibody clone suffix is shown within brackets.

Antibody	*q*-value
PDAC-NPC-OPD	

GAK (3)	1.21*E* − 45
IL-6 (7)	5.86*E* − 41
GAK (2)	3.18*E* − 34
IL-11 (2)	1.30*E* − 30
LDL (2)	3.57*E* − 30
TNF-*α* (3)	9.22*E* − 30
Procathepsin W	2.04*E* − 25
IL-13 (3)	2.98*E* − 25
MAPK8 (1)	9.85*E* − 21
IL-1*α* (1)	1.71*E* − 18
IL-13 (2)	8.94*E* − 18
TNFRSF3 (1)	2.08*E* − 14
IL-18 (2)	1.20*E* − 13
IL-1ra (1)	2.02*E* − 13
HADH2 (3)	2.07*E* − 13
CD40 (1)	2.71*E* − 13
Cystatin C (4)	4.07*E* − 13
IL-4 (3)	5.08*E* − 13
CIMS (18)	1.06*E* − 12
CIMS (16)	1.06*E* − 12
VEGF (3)	1.19*E* − 12
FASN (3)	1.57*E* − 12
TGF-*β*1 (2)	3.19*E* − 12
CIMS (26)	3.19*E* − 12
CIMS (25)	7.18*E* − 12

PDAC versus NPC	

*Upregulated in PDAC*	
VEGF (3)	4.05*E* − 11
IL-1ra (1)	7.90*E* − 11
C1 inh. (1)	2.70*E* − 10
C1q	8.05*E* − 10
VEGF (1)	9.36*E* − 10
IL-16 (3)	9.36*E* − 10
CD40L	1.01*E* − 09
IL-4 (3)	1.36*E* − 09
Sialyl Lewis x	1.39*E* − 09
IL-18 (2)	1.57*E* − 09
CIMS (18)	1.90*E* − 09
CIMS (23)	3.36*E* − 09
MCP-1 (2)	3.76*E* − 09
CIMS (25)	4.03*E* − 09

*Downregulated in PDAC*	
GAK (3)	1.01*E* − 27
IL-6 (7)	1.62*E* − 25
GAK (1)	3.17*E* − 24
LDL (2)	8.45*E* − 21
MAPK8 (1)	2.48*E* − 18
IL-11 (2)	2.23*E* − 16
TNF-*α* (3)	1.23*E* − 14
HADH2 (3)	9.12*E* − 14
Procathepsin W	1.05*E* − 12
TNFRSF3 (1)	1.49*E* − 11
IL-13 (3)	5.72*E* − 10

PDAC versus OPD	

*Upregulated in PDAC*	
Cystatin C (3)	1.11*E* − 08
IL-13 (3)	1.59*E* − 08
IL-1*α* (1)	3.01*E* − 08
Surface ag X	2.84*E* − 07
BTK (2)	2.84*E* − 07
Cystatin C (4)	2.84*E* − 07
CIMS (26)	2.84*E* − 07
CD40 (1)	3.75*E* − 07
TNFRSF3 (2)	4.19*E* − 07
ORP-3 (2)	6.34*E* − 07
Apo-A4 (3)	6.93*E* − 07
UPF3B (2)	6.93*E* − 07
MUC-1 (1)	7.87*E* − 07
TNF-*α* (3)	9.02*E* − 07
CIMS (16)	9.12*E* − 07
ATP-5B (1)	1.03*E* − 06
CIMS (12)	1.03*E* − 06
IL-13 (1)	1.03*E* − 06
MCP-1 (4)	1.49*E* − 06
CIMS (1)	1.67*E* − 06
CD40 (3)	1.79*E* − 06
Procathepsin W	1.90*E* − 06
TGF-*β*1 (2)	2.29*E* − 06
CIMS (24)	3.89*E* − 06
IL-18 (2)	5.59*E* − 06

**Table 3:** Consensus signatures of antibodies based on the highest combined score from 10 backward elimination iterations. Shown within brackets is the individual antibody clone suffix (for markers targeted by multiple antibody clones).

PDAC versus NPC	Score	PDAC versus OPD	Score
IL-11 (2)	291.4	MAPK1 (3)	276.5
IL-6 (7)	288.1	C5 (2)	273
Cystatin C (1)	286.9	TNFRSF3 (1)	265.5
C1 inh. (3)	279.2	TNFRSF3 (2)	260.9
Angiomotin (1)	276	UCHL5	259.6
IL-13 (2)	272.9	IL-4 (3)	258.7
IL-13 (3)	270.7	Factor B (3)	258
CD40 (1)	270.6	Apo-A4 (3)	257.5
HADH2 (3)	270.4	KSYK-1	255.2
HADH2 (4)	269.7	Sox11A	253.1
C1 inh. (4)	269.4	CD40L	252.2
C1 inh. (2)	269.2	Apo-A1 (1)	251.4
LDL (2)	268.1	CIMS (13)	250.1
GAK (3)	268	BTK (2)	246.1
C3 (1)	266.1	GM-CSF (5)	245
CIMS (5)	264.3	TGF-*β*1 (2)	239.5
C1q	261.1	PTP-1B (2)	237.2
CD40 (4)	259.6	MCP-1 (7)	235.1
IL-8 (2)	259.4	UPF3B (1)	232.5
C5 (2)	258.5	C1 inh. (4)	228.3
ATP-5B (3)	257.1	Sialyl Lewis x	227.6
Factor B (4)	256.2	IL-3 (1)	225.8
CIMS (10)	253.6	IL-9 (2)	224.2
TNF-*α* (3)	253.5	HADH2 (2)	222.7
Eotaxin (3)	248.4	IL-4 (4)	222.4
