# Supplementary material for: A Multicenter Trial Defining a Serum Protein Signature Associated with Pancreatic Ductal Adenocarcinoma
Source: Int J Proteomics. 2015 Oct 26;2015:587250. doi: 10.1155/2015/587250 (PMC4637476; doi:10.1155/2015/587250)
Supplement: Supplementary file 1 — Supplementary Figure 1: Slide and microarray lay-out. Supplementary Figure 2: Data visualized by principal component analysis. Supplementary Figure 3: Biomarker assessment by database queries for diseases by biomarkers. Supplementary Table 1: Antigens targeted on the antibody microarray. Supplementary Table 2: Context-independent motif specific antibodies used on the antibody microarrays. [file 587250.f1.pdf]

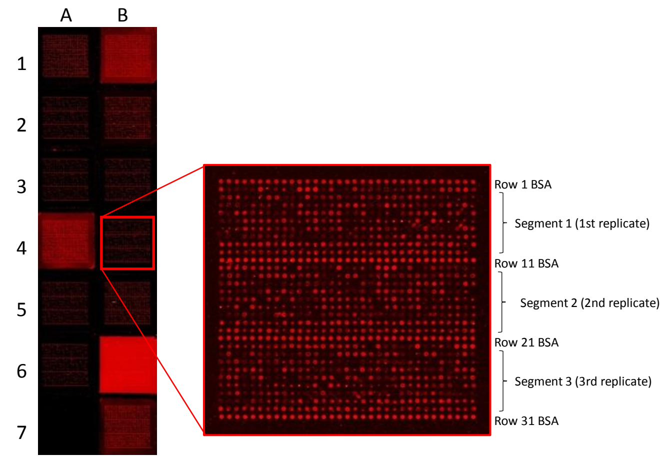

Supplement Figure 1. Slide and microarray lay-out. Thirteen identical subarrays were printed per slide (A1-6 and B1-7), each array consisting of 33x31 spots. Array B4 is enlarged to illustrate array design. Each antibody was printed in 3 replicates, placed in 3 different segments separated by rows of labeled BSA, and in different positions in each segment to assure reliable statistical measurement.

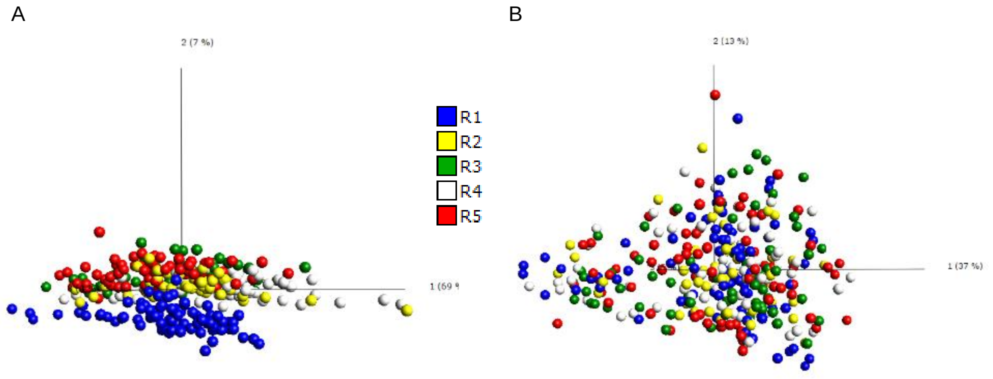

Supplement Figure 2. Principal component analysis of data colored according to the 5 different (consecutive) days/rounds (R1-R5) of analysis. A) Raw (logged) data showing technical batch-based differences. B) Normalized data in which the technical variation has been neutralized.

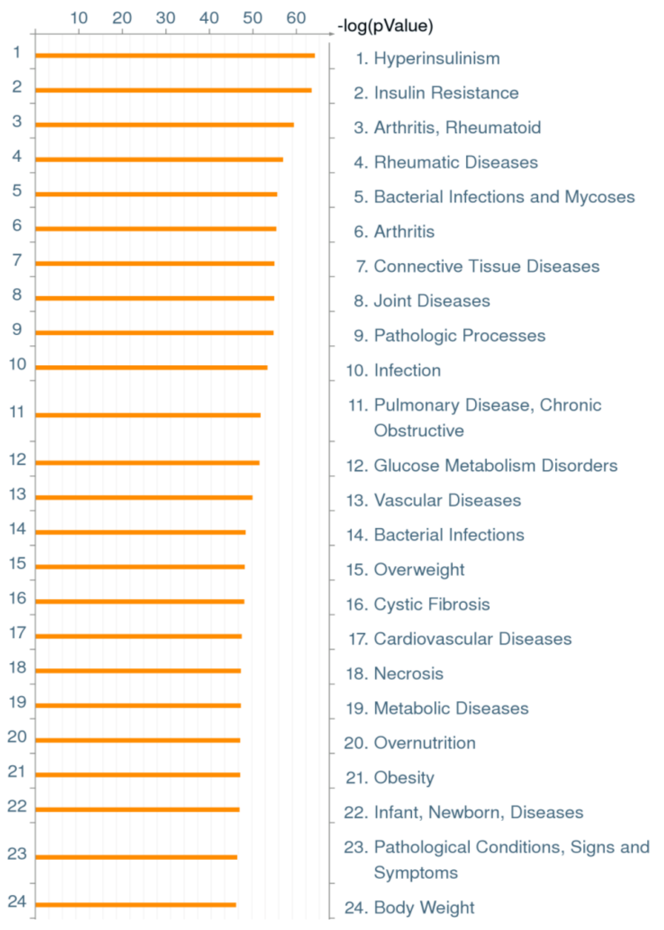

Supplement Figure 3. The top hits from pathway (Metacore) analysis when searching for diseases by biomarkers, using the entire set of antibodies and corresponding p-values and foldchanges for PDAC vs. NPC.

Supplement Table 1. Known antigens targeted on the antibody microarray

Protein	*Full name*	*No of antibody clones*
Angiomotin	Angiomotin	2
Apo-A1	Apolipoprotein A1	3
Apo-A4	Apolipoprotein A4	3
ATP-5B	ATP synthase subunit beta, mitochondrial	3
β-galactosidase	Beta-galactosidase	1
BTK	Tyrosine-protein kinase BTK	4
C1 inhibitor	Plasma protease C1 inhibitor	4
C1q*	Complement C1q	1
C1s	Complement C1s	1
C3*	Complement C3	6
C4*	Complement C4	4
C5*	Complement C5	3
CD40	CD40 protein	4
CD40L	CD40 ligand	1
CDK-2	Cyclin-dependent kinase 2	2
CHX10	Visual system homeobox 2	3
CT17	Cholera toxin subunit B	1
Cystatin C	Cystatin C	4
Digoxin	Digoxin	1
DUSP9	Dual specificity protein phosphatase 9	1
EGFR	Epidermal growth factor receptor	1
Eotaxin	Eotaxin	3
Factor B*	Complement factor B	4
FASN	Fatty acid synthase	4
GAK	Cyclin G-associated kinase	3
GLP-1	Glucagon-like peptide-1	1
GLP-1R	Glucagon-like peptide 1 receptor	1
GM-CSF	Granulocyte-macrophage colony-stimulating factor	6
HADH2	3-hydroxyacyl-CoA dehydrogenase type-2	4
Her2/ErbB-2	Receptor tyrosine-protein kinase erbB-2	4
HLA-DR/DP	HLA-DR/DP	1
ICAM-1	Intercellular adhesion molecule 1	1
IFN-γ	Interferon gamma	3
IgM	Immunoglobulin M	5
IL-10*	Interleukin-10	3
IL-11	Interleukin-11	3
IL-12*	Interleukin-12	4
IL-13*	Interleukin-13	3
IL-16	Interleukin-16	3
IL-18	Interleukin-18	3
IL-1α*	Interleukin-1 alpha	3
IL-1β	Interleukin-1 beta	3
IL-1ra	Interleukin-1 receptor antagonist protein	3
IL-2	Interleukin-2	3
IL-3	Interleukin-3	3
IL-4*	Interleukin-4	4
IL-5*	Interleukin-5	3
IL-6*	Interleukin-6	8
IL-7	Interleukin-7	2
IL-8*	Interleukin-8	3
IL-9	Interleukin-9	3
Integrin α-10	Integrin alpha-10	1
Integrin α-11	Integrin alpha-11	1
JAK3	Tyrosine-protein kinase JAK3	1
Keratin19	Keratin, type I cytoskeletal 19	3
KSYK	Tyrosine-protein kinase SYK	2
LDL	Apolipoprotein B-100	2
Leptin	Leptin	1
Lewis x	Lewis x	2
Lewis y	Lewis y	1
Lumican	Lumican	1
MAPK1	Mitogen-activated protein kinase 1	4
MAPK8	Mitogen-activated protein kinase 8	3
MATK	Megakaryocyte-associated tyrosine-protein kinase	3
MCP-1*	C-C motif chemokine 2	9
MCP-3	C-C motif chemokine 7	3
MCP-4	C-C motif chemokine 13	3
MUC-1	Mucin-1	6
Myomesin-2	Myomesin-2	2
ORP-3	Oxysterol-binding protein-related protein 3	2
Osteopontin	Osteopontin	3
P85A	Phosphatidylinositol 3-kinase regulatory subunit alpha	3
PKB gamma	RAC-gamma serine/threonine-protein kinase	2
Procathepsin W	Procathepsin W	1
Properdin*	Properdin	1
PSA	Prostate-specific antigen	1
PTK-6	Protein-tyrosine kinase 6	1
PTP-1B	Tyrosine-protein phosphatase non-receptor type 1	3
RANTES	C-C motif chemokine 5	3
RPS6KA2	Ribosomal protein S6 kinase alpha-2	3
Sialyl Lewis x	Sialyl Lewis x	1
Sox11A	Transcription factor SOX-11	1
STAP2	Signal-transducing adaptor protein 2	4
STAT1	Signal transducer and activator of transcription 1-alpha/beta	2
Surface Ag X	Surface Ag X	1
TBC1D9	TBC1 domain family member 9	3
TENS4	Tensin-4	1
TGF-β1	Transforming growth factor beta-1	3
TM peptide	Transmembrane peptide	1
TNF-α	Tumor necrosis factor	3
TNF-β*	Lymphotoxin-alpha	4
TNFRSF14	Tumor necrosis factor receptor superfamily member 14	2
TNFRSF3	Tumor necrosis factor receptor superfamily member 3	3
UBC9	SUMO-conjugating enzyme UBC9	3
UBE2C	Ubiquitin-conjugating enzyme E2 C	2
UCHL5	Ubiquitin carboxyl-terminal hydrolase isozyme L5	1
UPF3B	Regulator of nonsense transcripts 3B	2
VEGF*	Vascular endothelial growth factor	4

*Antibody specificity determined by protein arrays, MSD, ELISA, blocking/spiking experiments, and/or mass spectrometry

Supplement Table 2. Context-independent motif specific antibodies used on the antibody microarrays

CIMS antibody clone index	*Selection motif*
1	FLLMQYGGMDEHAR
2	AQQHQWDGLLSYQDSLS
3	GIVKYLYEDEG
4	GIVKYLYEDEG
5	WTRNSNMNYWLIIRL
6	WTRNSNMNYWLIIRL
7	LYEIAR
8	DFAEDK
9	LTEFAK
10	TEEQLK
11	TEEQLK
12	TEEQLK
13	SSAYSR
14	SSAYSR
15	EDFR
16	EDFR
17	SYVSLK
18	TLYVGK
19	EPFR
20	EPFR
21	LNVWGK
22	QEASFK
23	QEASFK
24	LSADHR
25	LSADHR
26	SEAHLR
27	SEAHLR
28	SEAHLR
29	SEAHLR
30	WDSR
31	WDSR

Supplement Table 3. Differential protein expression analysis of serum samples drawn from patients with differently located pancreatic tumors. Results are shown for body+tail tumors vs. head tumors, for the top 40 antibodies ($p<5\cdot10^{-5}$). FC = Fold change.

Antibody	*p-val*	*FC*
IL-1α (2)	4.32E-07	0.874447
CIMS (16)	1.28E-06	0.874676
VEGF (1)	1.47E-06	0.870962
TNF-β (2)	1.57E-06	0.872955
IL-11 (2)	2.15E-06	0.833144
CIMS (18)	3.09E-06	0.891206
CD40L	3.89E-06	0.917468
IL-3 (3)	4.19E-06	0.87497
CIMS (30)	4.88E-06	0.919455
IL-6 (1)	7.01E-06	0.873484
HLA-DR/DP	8.10E-06	0.879348
IL-2 (3)	8.34E-06	0.845869
Angiomotin (2)	8.45E-06	0.892806
Integrin α-10	9.07E-06	0.864779
IL-18 (3)	9.40E-06	0.899675
Sox11A	1.05E-05	0.898637
IL-7 (1)	1.18E-05	0.892816
MCP-1 (3)	1.20E-05	0.885187
Surface ag X	1.20E-05	0.865919
IL-9 (1)	1.21E-05	0.872639
CIMS (20)	1.36E-05	0.873286
IL-12 (3)	1.67E-05	0.872587
Lewis x (1)	1.77E-05	0.898973
IgM (3)	1.91E-05	0.893885
IL-7 (2)	1.98E-05	0.893886
CIMS (25)	2.25E-05	0.851688
CIMS (2)	2.30E-05	0.88583
CIMS (6)	2.41E-05	0.858863
IL-16 (3)	2.57E-05	0.905756
GLP-1	2.78E-05	0.890237
CHX10 (3)	2.83E-05	0.889864
IL-4 (3)	3.02E-05	0.860565
VEGF (4)	3.20E-05	0.848107
IL-3 (2)	3.35E-05	0.871146
IL-2 (2)	3.89E-05	0.843949
IL-1ra (3)	3.93E-05	0.88975
RANTES (3)	4.51E-05	0.933006
CIMS (31)	4.80E-05	0.886723
CIMS (27)	4.90E-05	0.915073
TGF-β1 (3)	4.91E-05	0.889071
